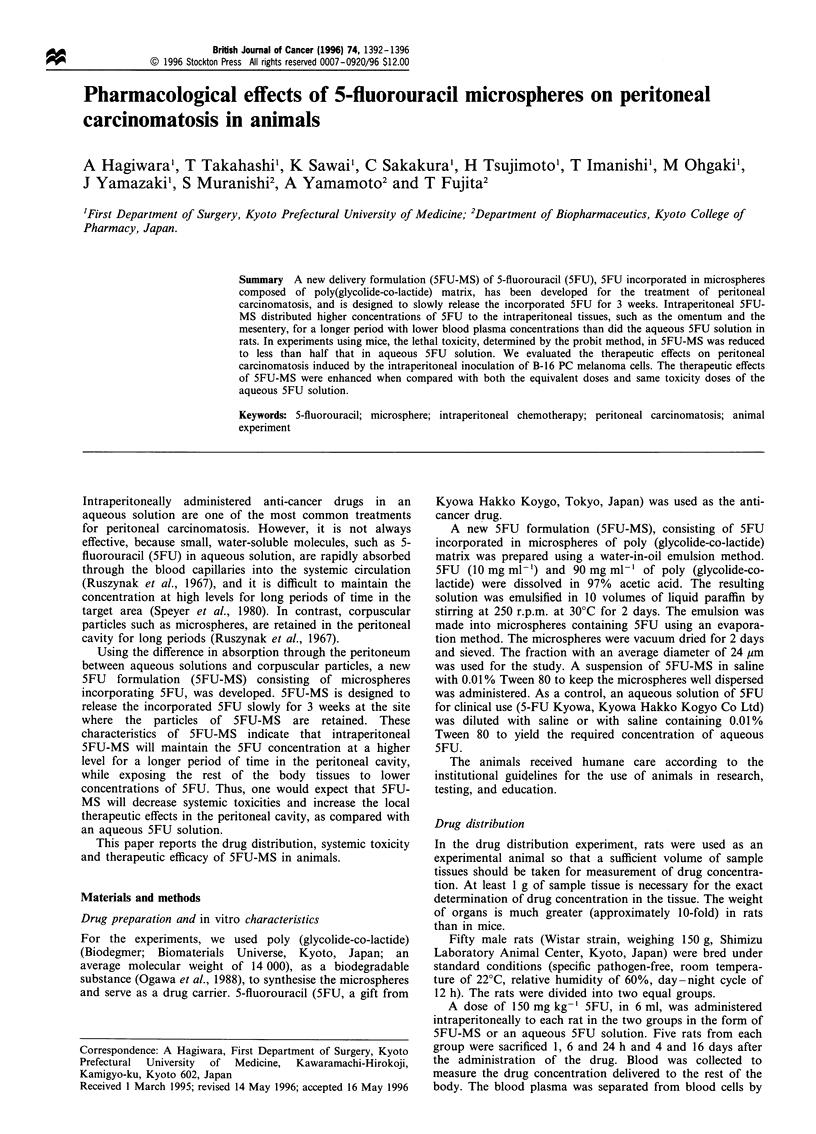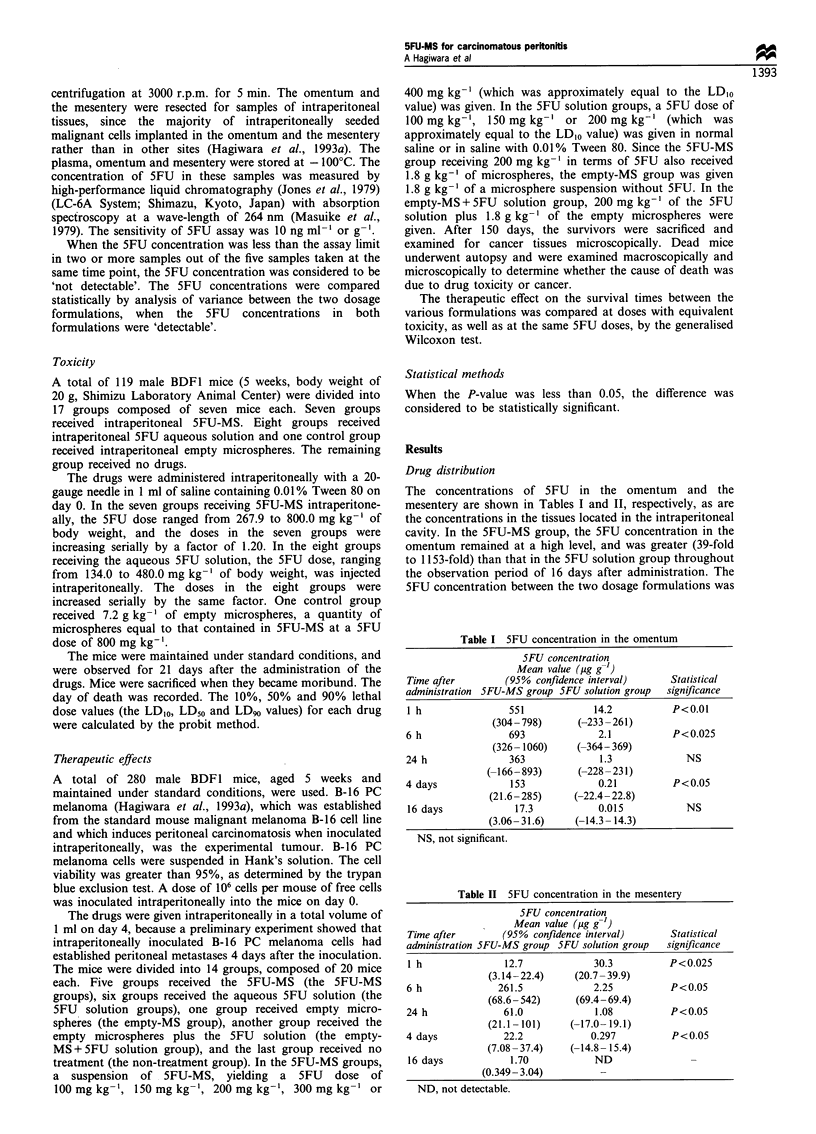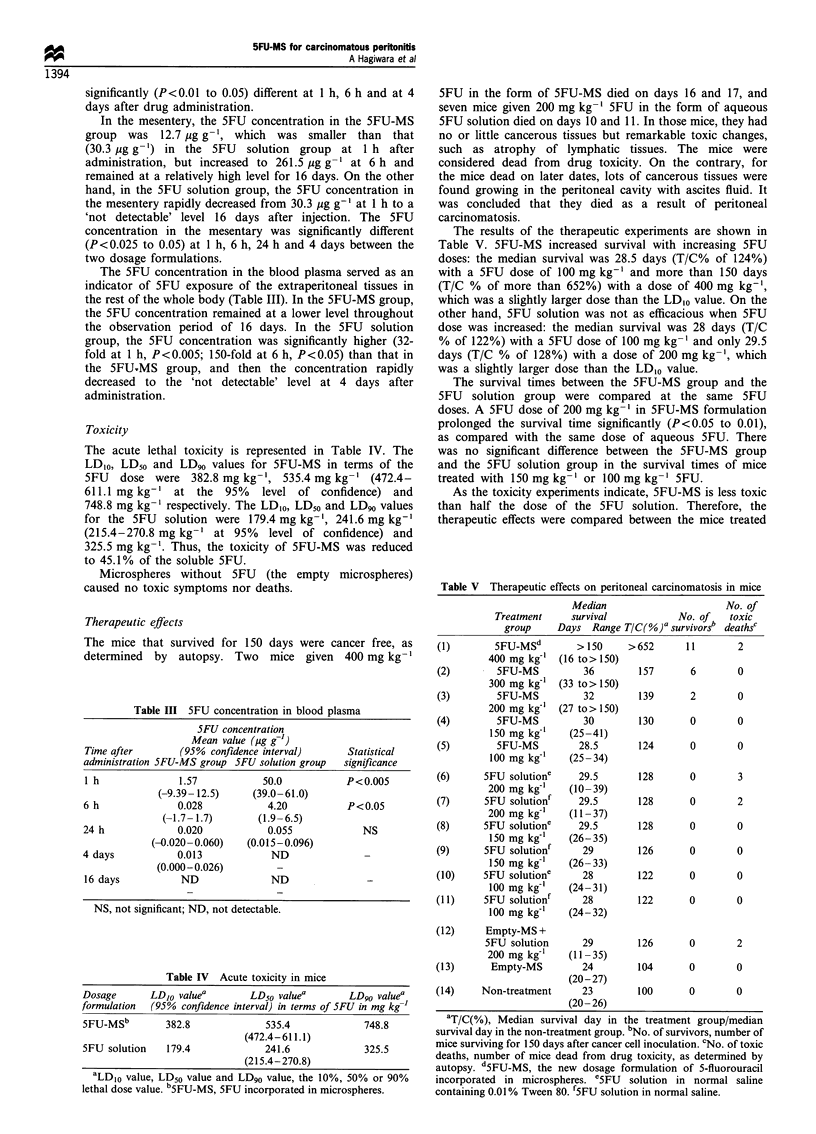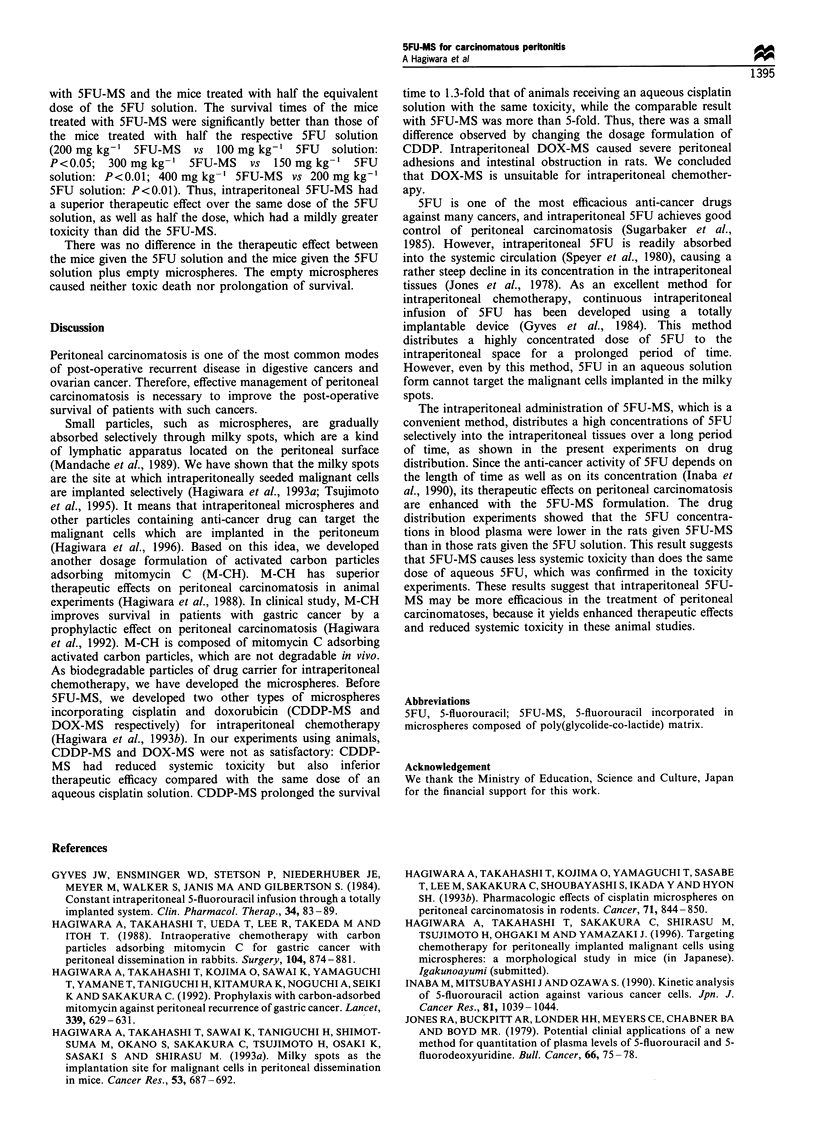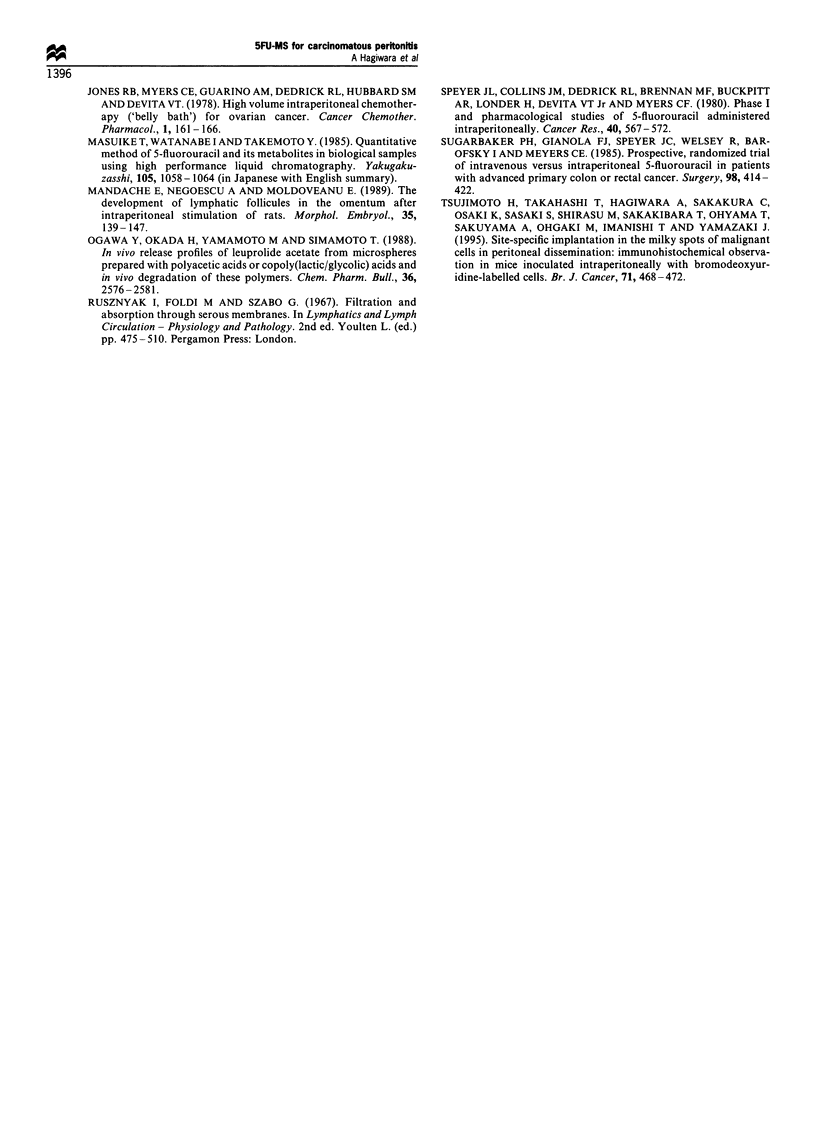# Pharmacological effects of 5-fluorouracil microspheres on peritoneal carcinomatosis in animals.

**DOI:** 10.1038/bjc.1996.554

**Published:** 1996-11

**Authors:** A. Hagiwara, T. Takahashi, K. Sawai, C. Sakakura, H. Tsujimoto, T. Imanishi, M. Ohgaki, J. Yamazaki, S. Muranishi, A. Yamamoto, T. Fujita

**Affiliations:** First Department of Surgery, Kyoto Prefectural University of Medicine, Japan.

## Abstract

A new delivery formulation (5FU-MS) of 5-fluorouracil (5FU), 5FU incorporated in microspheres composed of poly(glycolide-co-lactide) matrix, has been developed for the treatment of peritoneal carcinomatosis, and is designed to slowly release the incorporated 5FU for 3 weeks. Intraperitoneal 5FU-MS distributed higher concentrations of 5FU to the intraperitoneal tissues, such as the omentum and the mesentery, for a longer period with lower blood plasma concentrations than did the aqueous 5FU solution in rats. In experiments using mice, the lethal toxicity, determined by the probit method, in 5FU-MS was reduced to less than half that in aqueous 5FU solution. We evaluated the therapeutic effects on peritoneal carcinomatosis induced by the intraperitoneal inoculation of B-16 PC melanoma cells. The therapeutic effects of 5FU-MS were enhanced when compared with both the equivalent doses and same toxicity doses of the aqueous 5FU solution.


British Journal of Cancer (1996) 74, 1392-1396
© 1996 Stockton Press All rights reserved 0007-0920/96 $12.00

### Pharmacological effects of 5-fluorouracil microspheres on peritoneal carcinomatosis in animals

A Hagiwara[1], T Takahashi[1], K Sawai[1], C Sakakura[1], H Tsujimoto[1], T Imanishi[1], M Ohgaki[1], J Yamazaki[1], S Muranishi[2], A Yamamoto[2] and T Fujita[2]

[1]*First Department of Surgery, Kyoto Prefectural University of Medicine;* [2]*Department of Biopharmaceutics, Kyoto College of Pharmacy, Japan.*

**Summary** A new delivery formulation (5FU-MS) of 5-fluorouracil (5FU), 5FU incorporated in microspheres composed of poly(glycolide-co-lactide) matrix, has been developed for the treatment of peritoneal carcinomatosis, and is designed to slowly release the incorporated 5FU for 3 weeks. Intraperitoneal 5FU-MS distributed higher concentrations of 5FU to the intraperitoneal tissues, such as the omentum and the mesentery, for a longer period with lower blood plasma concentrations than did the aqueous 5FU solution in rats. In experiments using mice, the lethal toxicity, determined by the probit method, in 5FU-MS was reduced to less than half that in aqueous 5FU solution. We evaluated the therapeutic effects on peritoneal carcinomatosis induced by the intraperitoneal inoculation of B-16 PC melanoma cells. The therapeutic effects of 5FU-MS were enhanced when compared with both the equivalent doses and same toxicity doses of the aqueous 5FU solution.

**Keywords:** 5-fluorouracil; microsphere; intraperitoneal chemotherapy; peritoneal carcinomatosis; animal experiment

Intraperitoneally administered anti-cancer drugs in an aqueous solution are one of the most common treatments for peritoneal carcinomatosis. However, it is not always effective, because small, water-soluble molecules, such as 5-fluorouracil (5FU) in aqueous solution, are rapidly absorbed through the blood capillaries into the systemic circulation (Ruszynak *et al.*, 1967), and it is difficult to maintain the concentration at high levels for long periods of time in the target area (Speyer *et al.*, 1980). In contrast, corpuscular particles such as microspheres, are retained in the peritoneal cavity for long periods (Ruszynak *et al.*, 1967).

Using the difference in absorption through the peritoneum between aqueous solutions and corpuscular particles, a new 5FU formulation (5FU-MS) consisting of microspheres incorporating 5FU, was developed. 5FU-MS is designed to release the incorporated 5FU slowly for 3 weeks at the site where the particles of 5FU-MS are retained. These characteristics of 5FU-MS indicate that intraperitoneal 5FU-MS will maintain the 5FU concentration at a higher level for a longer period of time in the peritoneal cavity, while exposing the rest of the body tissues to lower concentrations of 5FU. Thus, one would expect that 5FU-MS will decrease systemic toxicities and increase the local therapeutic effects in the peritoneal cavity, as compared with an aqueous 5FU solution.

This paper reports the drug distribution, systemic toxicity and therapeutic efficacy of 5FU-MS in animals.

### Materials and methods

### *Drug preparation and* in vitro *characteristics*

For the experiments, we used poly (glycolide-co-lactide) (Biodegmer; Biomaterials Universe, Kyoto, Japan; an average molecular weight of 14 000), as a biodegradable substance (Ogawa *et al.*, 1988), to synthesise the microspheres and serve as a drug carrier. 5-fluorouracil (5FU, a gift from Kyowa Hakko Koygo, Tokyo, Japan) was used as the anti-cancer drug.

A new 5FU formulation (5FU-MS), consisting of 5FU incorporated in microspheres of poly (glycolide-co-lactide) matrix was prepared using a water-in-oil emulsion method. 5FU (10 mg $ml^{-1}$) and 90 mg $ml^{-1}$ of poly (glycolide-co-lactide) were dissolved in 97% acetic acid. The resulting solution was emulsified in 10 volumes of liquid paraffin by stirring at 250 r.p.m. at 30°C for 2 days. The emulsion was made into microspheres containing 5FU using an evaporation method. The microspheres were vacuum dried for 2 days and sieved. The fraction with an average diameter of 24 $\mu$m was used for the study. A suspension of 5FU-MS in saline with 0.01% Tween 80 to keep the microspheres well dispersed was administered. As a control, an aqueous solution of 5FU for clinical use (5-FU Kyowa, Kyowa Hakko Kogyo Co Ltd) was diluted with saline or with saline containing 0.01% Tween 80 to yield the required concentration of aqueous 5FU.

The animals received humane care according to the institutional guidelines for the use of animals in research, testing, and education.

### *Drug distribution*

In the drug distribution experiment, rats were used as an experimental animal so that a sufficient volume of sample tissues should be taken for measurement of drug concentration. At least 1 g of sample tissue is necessary for the exact determination of drug concentration in the tissue. The weight of organs is much greater (approximately 10-fold) in rats than in mice.

Fifty male rats (Wistar strain, weighing 150 g, Shimizu Laboratory Animal Center, Kyoto, Japan) were bred under standard conditions (specific pathogen-free, room temperature of 22°C, relative humidity of 60%, day–night cycle of 12 h). The rats were divided into two equal groups.

A dose of 150 mg $kg^{-1}$ 5FU, in 6 ml, was administered intraperitoneally to each rat in the two groups in the form of 5FU-MS or an aqueous 5FU solution. Five rats from each group were sacrificed 1, 6 and 24 h and 4 and 16 days after the administration of the drug. Blood was collected to measure the drug concentration delivered to the rest of the body. The blood plasma was separated from blood cells by

Correspondence: A Hagiwara, First Department of Surgery, Kyoto Prefectural University of Medicine, Kawaramachi-Hirokoji, Kamigyo-ku, Kyoto 602, Japan
Received 1 March 1995; revised 14 May 1996; accepted 16 May 1996

centrifugation at 3000 r.p.m. for 5 min. The omentum and the mesentery were resected for samples of intraperitoneal tissues, since the majority of intraperitoneally seeded malignant cells implanted in the omentum and the mesentery rather than in other sites (Hagiwara *et al.*, 1993*a*). The plasma, omentum and mesentery were stored at −100°C. The concentration of 5FU in these samples was measured by high-performance liquid chromatography (Jones *et al.*, 1979) (LC-6A System; Shimazu, Kyoto, Japan) with absorption spectroscopy at a wave-length of 264 nm (Masuike *et al.*, 1979). The sensitivity of 5FU assay was 10 ng ml$^{-1}$ or g$^{-1}$.

When the 5FU concentration was less than the assay limit in two or more samples out of the five samples taken at the same time point, the 5FU concentration was considered to be 'not detectable'. The 5FU concentrations were compared statistically by analysis of variance between the two dosage formulations, when the 5FU concentrations in both formulations were 'detectable'.

### *Toxicity*

A total of 119 male BDF1 mice (5 weeks, body weight of 20 g, Shimizu Laboratory Animal Center) were divided into 17 groups composed of seven mice each. Seven groups received intraperitoneal 5FU-MS. Eight groups received intraperitoneal 5FU aqueous solution and one control group received intraperitoneal empty microspheres. The remaining group received no drugs.

The drugs were administered intraperitoneally with a 20-gauge needle in 1 ml of saline containing 0.01% Tween 80 on day 0. In the seven groups receiving 5FU-MS intraperitoneally, the 5FU dose ranged from 267.9 to 800.0 mg kg$^{-1}$ of body weight, and the doses in the seven groups were increasing serially by a factor of 1.20. In the eight groups receiving the aqueous 5FU solution, the 5FU dose, ranging from 134.0 to 480.0 mg kg$^{-1}$ of body weight, was injected intraperitoneally. The doses in the eight groups were increased serially by the same factor. One control group received 7.2 g kg$^{-1}$ of empty microspheres, a quantity of microspheres equal to that contained in 5FU-MS at a 5FU dose of 800 mg kg$^{-1}$.

The mice were maintained under standard conditions, and were observed for 21 days after the administration of the drugs. Mice were sacrificed when they became moribund. The day of death was recorded. The 10%, 50% and 90% lethal dose values (the $LD_{10}$, $LD_{50}$ and $LD_{90}$ values) for each drug were calculated by the probit method.

### *Therapeutic effects*

A total of 280 male BDF1 mice, aged 5 weeks and maintained under standard conditions, were used. B-16 PC melanoma (Hagiwara *et al.*, 1993*a*), which was established from the standard mouse malignant melanoma B-16 cell line and which induces peritoneal carcinomatosis when inoculated intraperitoneally, was the experimental tumour. B-16 PC melanoma cells were suspended in Hank's solution. The cell viability was greater than 95%, as determined by the trypan blue exclusion test. A dose of $10^6$ cells per mouse of free cells was inoculated intraperitoneally into the mice on day 0.

The drugs were given intraperitoneally in a total volume of 1 ml on day 4, because a preliminary experiment showed that intraperitoneally inoculated B-16 PC melanoma cells had established peritoneal metastases 4 days after the inoculation. The mice were divided into 14 groups, composed of 20 mice each. Five groups received the 5FU-MS (the 5FU-MS groups), six groups received the aqueous 5FU solution (the 5FU solution groups), one group received empty microspheres (the empty-MS group), another group received the empty microspheres plus the 5FU solution (the empty-MS + 5FU solution group), and the last group received no treatment (the non-treatment group). In the 5FU-MS groups, a suspension of 5FU-MS, yielding a 5FU dose of 100 mg kg$^{-1}$, 150 mg kg$^{-1}$, 200 mg kg$^{-1}$, 300 mg kg$^{-1}$ or 400 mg kg$^{-1}$ (which was approximately equal to the $LD_{10}$ value) was given. In the 5FU solution groups, a 5FU dose of 100 mg kg$^{-1}$, 150 mg kg$^{-1}$ or 200 mg kg$^{-1}$ (which was approximately equal to the $LD_{10}$ value) was given in normal saline or in saline with 0.01% Tween 80. Since the 5FU-MS group receiving 200 mg kg$^{-1}$ in terms of 5FU also received 1.8 g kg$^{-1}$ of microspheres, the empty-MS group was given 1.8 g kg$^{-1}$ of a microsphere suspension without 5FU. In the empty-MS + 5FU solution group, 200 mg kg$^{-1}$ of the 5FU solution plus 1.8 g kg$^{-1}$ of the empty microspheres were given. After 150 days, the survivors were sacrificed and examined for cancer tissues microscopically. Dead mice underwent autopsy and were examined macroscopically and microscopically to determine whether the cause of death was due to drug toxicity or cancer.

The therapeutic effect on the survival times between the various formulations was compared at doses with equivalent toxicity, as well as at the same 5FU doses, by the generalised Wilcoxon test.

### *Statistical methods*

When the *P*-value was less than 0.05, the difference was considered to be statistically significant.

### Results

### *Drug distribution*

The concentrations of 5FU in the omentum and the mesentery are shown in Tables I and II, respectively, as are the concentrations in the tissues located in the intraperitoneal cavity. In the 5FU-MS group, the 5FU concentration in the omentum remained at a high level, and was greater (39-fold to 1153-fold) than that in the 5FU solution group throughout the observation period of 16 days after administration. The 5FU concentration between the two dosage formulations was

**Table I** 5FU concentration in the omentum

| Time after administration | 5FU concentration Mean value (μg g$^{-1}$) (95% confidence interval) 5FU-MS group | 5FU solution group | Statistical significance |
|---|---|---|---|
| 1 h | 551 (304–798) | 14.2 (−233–261) | $P<0.01$ |
| 6 h | 693 (326–1060) | 2.1 (−364–369) | $P<0.025$ |
| 24 h | 363 (−166–893) | 1.3 (−228–231) | NS |
| 4 days | 153 (21.6–285) | 0.21 (−22.4–22.8) | $P<0.05$ |
| 16 days | 17.3 (3.06–31.6) | 0.015 (−14.3–14.3) | NS |

NS, not significant.

**Table II** 5FU concentration in the mesentery

| Time after administration | 5FU concentration Mean value (μg g$^{-1}$) (95% confidence interval) 5FU-MS group | 5FU solution group | Statistical significance |
|---|---|---|---|
| 1 h | 12.7 (3.14–22.4) | 30.3 (20.7–39.9) | $P<0.025$ |
| 6 h | 261.5 (68.6–542) | 2.25 (69.4–69.4) | $P<0.05$ |
| 24 h | 61.0 (21.1–101) | 1.08 (−17.0–19.1) | $P<0.05$ |
| 4 days | 22.2 (7.08–37.4) | 0.297 (−14.8–15.4) | $P<0.05$ |
| 16 days | 1.70 (0.349–3.04) | ND – | – |

ND, not detectable.

significantly ($P<0.01$ to 0.05) different at 1 h, 6 h and at 4 days after drug administration.

In the mesentery, the 5FU concentration in the 5FU-MS group was 12.7 $\mu g\ g^{-1}$, which was smaller than that (30.3 $\mu g\ g^{-1}$) in the 5FU solution group at 1 h after administration, but increased to 261.5 $\mu g\ g^{-1}$ at 6 h and remained at a relatively high level for 16 days. On the other hand, in the 5FU solution group, the 5FU concentration in the mesentery rapidly decreased from 30.3 $\mu g\ g^{-1}$ at 1 h to a 'not detectable' level 16 days after injection. The 5FU concentration in the mesentary was significantly different ($P<0.025$ to 0.05) at 1 h, 6 h, 24 h and 4 days between the two dosage formulations.

The 5FU concentration in the blood plasma served as an indicator of 5FU exposure of the extraperitoneal tissues in the rest of the whole body (Table III). In the 5FU-MS group, the 5FU concentration remained at a lower level throughout the observation period of 16 days. In the 5FU solution group, the 5FU concentration was significantly higher (32-fold at 1 h, $P<0.005$; 150-fold at 6 h, $P<0.05$) than that in the 5FU-MS group, and then the concentration rapidly decreased to the 'not detectable' level at 4 days after administration.

### *Toxicity*

The acute lethal toxicity is represented in Table IV. The $LD_{10}$, $LD_{50}$ and $LD_{90}$ values for 5FU-MS in terms of the 5FU dose were 382.8 mg $kg^{-1}$, 535.4 mg $kg^{-1}$ (472.4–611.1 mg $kg^{-1}$ at the 95% level of confidence) and 748.8 mg $kg^{-1}$ respectively. The $LD_{10}$, $LD_{50}$ and $LD_{90}$ values for the 5FU solution were 179.4 mg $kg^{-1}$, 241.6 mg $kg^{-1}$ (215.4–270.8 mg $kg^{-1}$ at 95% level of confidence) and 325.5 mg $kg^{-1}$. Thus, the toxicity of 5FU-MS was reduced to 45.1% of the soluble 5FU.

Microspheres without 5FU (the empty microspheres) caused no toxic symptoms nor deaths.

### *Therapeutic effects*

The mice that survived for 150 days were cancer free, as determined by autopsy. Two mice given 400 mg $kg^{-1}$ 5FU in the form of 5FU-MS died on days 16 and 17, and seven mice given 200 mg $kg^{-1}$ 5FU in the form of aqueous 5FU solution died on days 10 and 11. In those mice, they had no or little cancerous tissues but remarkable toxic changes, such as atrophy of lymphatic tissues. The mice were considered dead from drug toxicity. On the contrary, for the mice dead on later dates, lots of cancerous tissues were found growing in the peritoneal cavity with ascites fluid. It was concluded that they died as a result of peritoneal carcinomatosis.

The results of the therapeutic experiments are shown in Table V. 5FU-MS increased survival with increasing 5FU doses: the median survival was 28.5 days (T/C% of 124%) with a 5FU dose of 100 mg $kg^{-1}$ and more than 150 days (T/C % of more than 652%) with a dose of 400 mg $kg^{-1}$, which was a slightly larger dose than the $LD_{10}$ value. On the other hand, 5FU solution was not as efficacious when 5FU dose was increased: the median survival was 28 days (T/C % of 122%) with a 5FU dose of 100 mg $kg^{-1}$ and only 29.5 days (T/C % of 128%) with a dose of 200 mg $kg^{-1}$, which was a slightly larger dose than the $LD_{10}$ value.

The survival times between the 5FU-MS group and the 5FU solution group were compared at the same 5FU doses. A 5FU dose of 200 mg $kg^{-1}$ in 5FU-MS formulation prolonged the survival time significantly ($P<0.05$ to 0.01), as compared with the same dose of aqueous 5FU. There was no significant difference between the 5FU-MS group and the 5FU solution group in the survival times of mice treated with 150 mg $kg^{-1}$ or 100 mg $kg^{-1}$ 5FU.

As the toxicity experiments indicate, 5FU-MS is less toxic than half the dose of the 5FU solution. Therefore, the therapeutic effects were compared between the mice treated

**Table III** 5FU concentration in blood plasma

| *Time after administration* | *5FU concentration Mean value ($\mu g\ g^{-1}$) (95% confidence interval)* 5FU-MS group | 5FU solution group | *Statistical significance* |
|---|---|---|---|
| 1 h | 1.57 (–9.39–12.5) | 50.0 (39.0–61.0) | $P<0.005$ |
| 6 h | 0.028 (–1.7–1.7) | 4.20 (1.9–6.5) | $P<0.05$ |
| 24 h | 0.020 (–0.020–0.060) | 0.055 (0.015–0.096) | NS |
| 4 days | 0.013 (0.000–0.026) | ND – | – |
| 16 days | ND – | ND – | – |

NS, not significant; ND, not detectable.

**Table IV** Acute toxicity in mice

| *Dosage formulation* | *$LD_{10}$ value[a]* | *$LD_{50}$ value[a]* | *$LD_{90}$ value[a]* |
|---|---|---|---|
| | *(95% confidence interval) in terms of 5FU in mg $kg^{-1}$* | | |
| 5FU-MS[b] | 382.8 | 535.4 (472.4–611.1) | 748.8 |
| 5FU solution | 179.4 | 241.6 (215.4–270.8) | 325.5 |

[a]$LD_{10}$ value, $LD_{50}$ value and $LD_{90}$ value, the 10%, 50% or 90% lethal dose value. [b]5FU-MS, 5FU incorporated in microspheres.

**Table V** Therapeutic effects on peritoneal carcinomatosis in mice

| | *Treatment group* | *Median survival Days* | *Range* | *T/C(%)[a]* | *No. of survivors[b]* | *No. of toxic deaths[c]* |
|---|---|---|---|---|---|---|
| (1) | 5FU-MS[d] 400 mg $kg^{-1}$ | >150 | (16 to >150) | >652 | 11 | 2 |
| (2) | 5FU-MS 300 mg $kg^{-1}$ | 36 | (33 to >150) | 157 | 6 | 0 |
| (3) | 5FU-MS 200 mg $kg^{-1}$ | 32 | (27 to >150) | 139 | 2 | 0 |
| (4) | 5FU-MS 150 mg $kg^{-1}$ | 30 | (25–41) | 130 | 0 | 0 |
| (5) | 5FU-MS 100 mg $kg^{-1}$ | 28.5 | (25–34) | 124 | 0 | 0 |
| (6) | 5FU solution[e] 200 mg $kg^{-1}$ | 29.5 | (10–39) | 128 | 0 | 3 |
| (7) | 5FU solution[f] 200 mg $kg^{-1}$ | 29.5 | (11–37) | 128 | 0 | 2 |
| (8) | 5FU solution[e] 150 mg $kg^{-1}$ | 29.5 | (26–35) | 128 | 0 | 0 |
| (9) | 5FU solution[f] 150 mg $kg^{-1}$ | 29 | (26–33) | 126 | 0 | 0 |
| (10) | 5FU solution[e] 100 mg $kg^{-1}$ | 28 | (24–31) | 122 | 0 | 0 |
| (11) | 5FU solution[f] 100 mg $kg^{-1}$ | 28 | (24–32) | 122 | 0 | 0 |
| (12) | Empty-MS + 5FU solution 200 mg $kg^{-1}$ | 29 | (11–35) | 126 | 0 | 2 |
| (13) | Empty-MS | 24 | (20–27) | 104 | 0 | 0 |
| (14) | Non-treatment | 23 | (20–26) | 100 | 0 | 0 |

[a]T/C(%), Median survival day in the treatment group/median survival day in the non-treatment group. [b]No. of survivors, number of mice surviving for 150 days after cancer cell inoculation. [c]No. of toxic deaths, number of mice dead from drug toxicity, as determined by autopsy. [d]5FU-MS, the new dosage formulation of 5-fluorouracil incorporated in microspheres. [e]5FU solution in normal saline containing 0.01% Tween 80. [f]5FU solution in normal saline.

with 5FU-MS and the mice treated with half the equivalent dose of the 5FU solution. The survival times of the mice treated with 5FU-MS were significantly better than those of the mice treated with half the respective 5FU solution (200 mg $kg^{-1}$ 5FU-MS *vs* 100 mg $kg^{-1}$ 5FU solution: $P<0.05$; 300 mg $kg^{-1}$ 5FU-MS *vs* 150 mg $kg^{-1}$ 5FU solution: $P<0.01$; 400 mg $kg^{-1}$ 5FU-MS *vs* 200 mg $kg^{-1}$ 5FU solution: $P<0.01$). Thus, intraperitoneal 5FU-MS had a superior therapeutic effect over the same dose of the 5FU solution, as well as half the dose, which had a mildly greater toxicity than did the 5FU-MS.

There was no difference in the therapeutic effect between the mice given the 5FU solution and the mice given the 5FU solution plus empty microspheres. The empty microspheres caused neither toxic death nor prolongation of survival.

### Discussion

Peritoneal carcinomatosis is one of the most common modes of post-operative recurrent disease in digestive cancers and ovarian cancer. Therefore, effective management of peritoneal carcinomatosis is necessary to improve the post-operative survival of patients with such cancers.

Small particles, such as microspheres, are gradually absorbed selectively through milky spots, which are a kind of lymphatic apparatus located on the peritoneal surface (Mandache *et al.*, 1989). We have shown that the milky spots are the site at which intraperitoneally seeded malignant cells are implanted selectively (Hagiwara *et al.*, 1993*a*; Tsujimoto *et al.*, 1995). It means that intraperitoneal microspheres and other particles containing anti-cancer drug can target the malignant cells which are implanted in the peritoneum (Hagiwara *et al.*, 1996). Based on this idea, we developed another dosage formulation of activated carbon particles adsorbing mitomycin C (M-CH). M-CH has superior therapeutic effects on peritoneal carcinomatosis in animal experiments (Hagiwara *et al.*, 1988). In clinical study, M-CH improves survival in patients with gastric cancer by a prophylactic effect on peritoneal carcinomatosis (Hagiwara *et al.*, 1992). M-CH is composed of mitomycin C adsorbing activated carbon particles, which are not degradable *in vivo*. As biodegradable particles of drug carrier for intraperitoneal chemotherapy, we have developed the microspheres. Before 5FU-MS, we developed two other types of microspheres incorporating cisplatin and doxorubicin (CDDP-MS and DOX-MS respectively) for intraperitoneal chemotherapy (Hagiwara *et al.*, 1993*b*). In our experiments using animals, CDDP-MS and DOX-MS were not as satisfactory: CDDP-MS had reduced systemic toxicity but also inferior therapeutic efficacy compared with the same dose of an aqueous cisplatin solution. CDDP-MS prolonged the survival time to 1.3-fold that of animals receiving an aqueous cisplatin solution with the same toxicity, while the comparable result with 5FU-MS was more than 5-fold. Thus, there was a small difference observed by changing the dosage formulation of CDDP. Intraperitoneal DOX-MS caused severe peritoneal adhesions and intestinal obstruction in rats. We concluded that DOX-MS is unsuitable for intraperitoneal chemotherapy.

5FU is one of the most efficacious anti-cancer drugs against many cancers, and intraperitoneal 5FU achieves good control of peritoneal carcinomatosis (Sugarbaker *et al.*, 1985). However, intraperitoneal 5FU is readily absorbed into the systemic circulation (Speyer *et al.*, 1980), causing a rather steep decline in its concentration in the intraperitoneal tissues (Jones *et al.*, 1978). As an excellent method for intraperitoneal chemotherapy, continuous intraperitoneal infusion of 5FU has been developed using a totally implantable device (Gyves *et al.*, 1984). This method distributes a highly concentrated dose of 5FU to the intraperitoneal space for a prolonged period of time. However, even by this method, 5FU in an aqueous solution form cannot target the malignant cells implanted in the milky spots.

The intraperitoneal administration of 5FU-MS, which is a convenient method, distributes a high concentrations of 5FU selectively into the intraperitoneal tissues over a long period of time, as shown in the present experiments on drug distribution. Since the anti-cancer activity of 5FU depends on the length of time as well as on its concentration (Inaba *et al.*, 1990), its therapeutic effects on peritoneal carcinomatosis are enhanced with the 5FU-MS formulation. The drug distribution experiments showed that the 5FU concentrations in blood plasma were lower in the rats given 5FU-MS than in those rats given the 5FU solution. This result suggests that 5FU-MS causes less systemic toxicity than does the same dose of aqueous 5FU, which was confirmed in the toxicity experiments. These results suggest that intraperitoneal 5FU-MS may be more efficacious in the treatment of peritoneal carcinomatoses, because it yields enhanced therapeutic effects and reduced systemic toxicity in these animal studies.

### Abbreviations

5FU, 5-fluorouracil; 5FU-MS, 5-fluorouracil incorporated in microspheres composed of poly(glycolide-co-lactide) matrix.

### Acknowledgement

We thank the Ministry of Education, Science and Culture, Japan for the financial support for this work.

### References

GYVES JW, ENSMINGER WD, STETSON P, NIEDERHUBER JE, MEYER M, WALKER S, JANIS MA AND GILBERTSON S. (1984). Constant intraperitoneal 5-fluorouracil infusion through a totally implanted system. *Clin. Pharmacol. Therap.*, **34,** 83–89.

HAGIWARA A, TAKAHASHI T, UEDA T, LEE R, TAKEDA M AND ITOH T. (1988). Intraoperative chemotherapy with carbon particles adsorbing mitomycin C for gastric cancer with peritoneal dissemination in rabbits. *Surgery*, **104,** 874–881.

HAGIWARA A, TAKAHASHI T, KOJIMA O, SAWAI K, YAMAGUCHI T, YAMANE T, TANIGUCHI H, KITAMURA K, NOGUCHI A, SEIKI K AND SAKAKURA C. (1992). Prophylaxis with carbon-adsorbed mitomycin against peritoneal recurrence of gastric cancer. *Lancet*, **339,** 629–631.

HAGIWARA A, TAKAHASHI T, SAWAI K, TANIGUCHI H, SHIMOTSUMA M, OKANO S, SAKAKURA C, TSUJIMOTO H, OSAKI K, SASAKI S AND SHIRASU M. (1993*a*). Milky spots as the implantation site for malignant cells in peritoneal dissemination in mice. *Cancer Res.*, **53,** 687–692.

HAGIWARA A, TAKAHASHI T, KOJIMA O, YAMAGUCHI T, SASABE T, LEE M, SAKAKURA C, SHOUBAYASHI S, IKADA Y AND HYON SH. (1993*b*). Pharmacologic effects of cisplatin microspheres on peritoneal carcinomatosis in rodents. *Cancer*, **71,** 844–850.

HAGIWARA A, TAKAHASHI T, SAKAKURA C, SHIRASU M, TSUJIMOTO H, OHGAKI M AND YAMAZAKI J. (1996). Targeting chemotherapy for peritoneally implanted malignant cells using microspheres: a morphological study in mice (in Japanese). *Igakunoayumi* (submitted).

INABA M, MITSUBAYASHI J AND OZAWA S. (1990). Kinetic analysis of 5-fluorouracil action against various cancer cells. *Jpn. J. Cancer Res.*, **81,** 1039–1044.

JONES RA, BUCKPITT AR, LONDER HH, MEYERS CE, CHABNER BA AND BOYD MR. (1979). Potential clinial applications of a new method for quantitation of plasma levels of 5-fluorouracil and 5-fluorodeoxyuridine. *Bull. Cancer*, **66,** 75–78.

JONES RB, MYERS CE, GUARINO AM, DEDRICK RL, HUBBARD SM AND DEVITA VT. (1978). High volume intraperitoneal chemotherapy ('belly bath') for ovarian cancer. *Cancer Chemother. Pharmacol.*, **1**, 161-166.

MASUIKE T, WATANABE I AND TAKEMOTO Y. (1985). Quantitative method of 5-fluorouracil and its metabolites in biological samples using high performance liquid chromatography. *Yakugaku-zasshi*, **105**, 1058-1064 (in Japanese with English summary).

MANDACHE E, NEGOESCU A AND MOLDOVEANU E. (1989). The development of lymphatic follicules in the omentum after intraperitoneal stimulation of rats. *Morphol. Embryol.*, **35**, 139-147.

OGAWA Y, OKADA H, YAMAMOTO M AND SIMAMOTO T. (1988). *In vivo* release profiles of leuprolide acetate from microspheres prepared with polyacetic acids or copoly(lactic/glycolic) acids and *in vivo* degradation of these polymers. *Chem. Pharm. Bull.*, **36**, 2576-2581.

RUSZNYAK I, FOLDI M AND SZABO G. (1967). Filtration and absorption through serous membranes. In *Lymphatics and Lymph Circulation - Physiology and Pathology*. 2nd ed. Youlten L. (ed.) pp. 475-510. Pergamon Press: London.

SPEYER JL, COLLINS JM, DEDRICK RL, BRENNAN MF, BUCKPITT AR, LONDER H, DEVITA VT JR AND MYERS CF. (1980). Phase I and pharmacological studies of 5-fluorouracil administered intraperitoneally. *Cancer Res.*, **40**, 567-572.

SUGARBAKER PH, GIANOLA FJ, SPEYER JC, WELSEY R, BAROFSKY I AND MEYERS CE. (1985). Prospective, randomized trial of intravenous versus intraperitoneal 5-fluorouracil in patients with advanced primary colon or rectal cancer. *Surgery*, **98**, 414-422.

TSUJIMOTO H, TAKAHASHI T, HAGIWARA A, SAKAKURA C, OSAKI K, SASAKI S, SHIRASU M, SAKAKIBARA T, OHYAMA T, SAKUYAMA A, OHGAKI M, IMANISHI T AND YAMAZAKI J. (1995). Site-specific implantation in the milky spots of malignant cells in peritoneal dissemination: immunohistochemical observation in mice inoculated intraperitoneally with bromodeoxyuridine-labelled cells. *Br. J. Cancer*, **71**, 468-472.